# Gynecological

**Published:** 1882-08-20

**Authors:** 


GYNECOLOGICAL.—We invite attention to the card of Drs. Taliaferro & Noble, in reference to their Infirmary, located in this city for the treatment of diseases of women. The Infirmary is pleasantly located, is commodious, airy and convenient—located adjacent to the Doctor's residence, and supplied with suitable appliances, nurses, etc. The general practitioner often finds it desireable, in obstinate cases, requiring special and unusual attention, to refer his patient to a specialist who is experienced and reliable, and who is provided with suitable appliances and conveniences for such cases.

---

THE attention of Doctors' wives is called to the advertisement of the Patti Hand Attachment to sewing machines in this issue.

